# Early and persisting increase in fat content after human muscle strain injuries in adults

**DOI:** 10.14814/phy2.71086

**Published:** 2026-09-01

**Authors:** Monika Lucia Bayer, Kenneth Hudlebusch Mertz, Asta Skovgaard Eriksen, Michael Kjaer, Stig Peter Magnusson, Frederik Hvid Linden, Rene Brüggebusch Svensson

**Affiliations:** ^1^ Department of Orthopedic Surgery M Institute of Sports Medicine Copenhagen, Copenhagen University Hospital, Bispebjerg Frederiksberg Copenhagen Denmark; ^2^ Department of Physical and Occupational Therapy Copenhagen University Hospital, Bispebjerg Frederiksberg Copenhagen Denmark; ^3^ Department of Radiology Copenhagen University Hospital, Bispebjerg Frederiksberg Copenhagen Denmark; ^4^ Department of Clinical Medicine, Faculty of Health and Medical Sciences University of Copenhagen Copenhagen Denmark; ^5^ Department of Health Technology, Center for Fast Ultrasound Imaging Technical University of Denmark Lyngby Denmark

**Keywords:** aponeurosis, fatty infiltration, muscle atrophy, muscle strain injury

## Abstract

Acute muscle strain injuries belong to the most frequent sports injuries with high recurrence rates. Biopsies obtained from previously strain‐injured muscles showed fat intra‐ and intermuscular, but quantification of fat content in the entire muscle volume following a strain injury has not been performed previously. The aim of the study was to evaluate fat fraction in the injured and the contralateral, uninjured muscle acutely, and 3‐ and 12‐months after a strain injury. DIXON fat fraction was analyzed acutely after the injury, 3‐ and 12‐months post injury in the injured and the contralateral, healthy muscle in 29 individuals with an ultrasound verified strain injury in either the calf or the hamstrings. There was a significant increase in fat content 3 and 12 months post injury in the injured, but not in the contralateral, uninjured muscles. There was a significant negative correlation between the increase in fat fraction and loss of muscle mass, and a significant positive correlation between the enlargement of the aponeurosis and the amount of fat in the injured muscles. Strain injuries lead to a rise in fat fraction in muscles from healthy, sports active individuals. The increased fat fraction appears permanent as fat accumulation in the muscle after the injury was not reversed after 12 months.

## 1 INTRODUCTION

Acute muscle strain injury is one of the most prevalent types of sport injuries in both professional and amateur athletes (Azar et al., 2024; Dahmen et al., 2023; Edouard et al., 2016; Ekstrand et al., 2023). In addition to the substantial injury prevalence, muscle strain injuries are also associated with one of the highest reinjury rates in sports (Wulff et al., 2024), often occurring at the same site as the index injury (Wangensteen et al., 2016). The significant injury recurrence suggests incomplete repair of the involved tissue structures, which is supported by findings of muscle strain injury leading to permanent loss of muscle mass (Mertz et al., 2025; Silder et al., 2008) and persistent inflammation (Bayer et al., 2019). Recent work on tissue biopsies from previously strain injured muscle reported fat accumulation intra‐myocellularly and inter‐myocellularly, which could not be reversed by 3 months of rehabilitation training (Bayer et al., 2021). While fat accumulation was observed years after a strain injury, the time course of fatty infiltration, especially in the early period following injury, is unknown. Further, biopsies represent only a tiny portion of the muscle and the amount of fat on a whole muscle level has not been quantified in strain injured muscles. Quantification of fat within muscle has benefitted from the introduction of a proton chemical‐shift imaging technique that can generate water‐only and fat‐only images from dual‐echo acquisition quantitative magnetic resonance imaging (MRI) such as DIXON (Dixon, 1984; Nardo et al., 2014). Fatty infiltration has been reported in a broad range of conditions, such as trauma to the rotator cuff (Kriechling et al., 2025; Wieser et al., 2019), muscular dystrophies (Batra et al., 2020; Marty et al., 2018), end stage osteoarthritis (Fitzgerald et al., 2023), aging (Delmonico et al., 2009) and metabolic diseases (Farup et al., 2021; Goodpaster et al., 2000). Fatty infiltration in skeletal muscle is one of the major contributors to impaired muscle quality, which again is a strong predictor of reduced muscle function and higher rates of recurrence and poor recovery, at least in rotator cuff tears (Godenèche et al., 2017; Kim et al., 2021). Whether there is an association between the amount of fatty infiltration following strain injuries and reinjury rate has remained unexplored.

Previous research has shown that muscle strain injuries not only affect muscle tissue, but also the connective tissue, that is, the aponeurosis (Lazarczuk et al., 2024; Nielsen et al., 2023). As a result to the injury, the aponeurosis becomes significantly enlarged (Mertz et al., 2025; Nielsen et al., 2023) and aponeurosis function appears altered as a consequence to a strain injury (Nielsen et al., 2023). Khair et al. reported recently an interesting correlation between muscle fascicle length and material properties of the aponeurosis determined by shear‐wave elastography in the medial gastrocnemius (Khair et al., 2025). This relationship suggests that changes in the muscle can lead to adaptations of the associated aponeurosis, or that the stiffness of the aponeurosis might impact muscle tissue properties. Whether the observed changes in aponeurosis structure might show an association with pathologic change of the muscle after a strain injury has remained unknown.

The present study used MRI to evaluate fat fraction of the entire muscle volume in the acute phase after the strain injury and at 3‐ and 12‐months post injury to gain a comprehensive overview of fat content in the muscle of sport active individuals after sustaining a strain injury. We hypothesized that there is an increased fat content during the time from the injury to the 3 months post scan with no improvement from 3 to 12 months.

## 2 MATERIALS AND METHODS

### 2.1 Participants

This study enrolled 50 sports‐active male and female individuals (participant details have been reported elsewhere (Mertz et al., 2025) and in the Table S1). Briefly, individuals with an acute muscle strain injury in the calf or hamstring musculature—defined as a sudden onset of pain and being unable to continue sports participation—were included. Participants were included based on clinical history (sudden onset of pain during explosive movement), pain on palpation of the suspected injured muscle(s), and a clear defect at the muscle‐connective tissue (aponeurosis) interface on an ultrasound scan. Exclusion criteria included claustrophobia (or other contraindications to MRI scans), daily intake of nonsteroidal anti‐inflammatory drugs (NSAIDs) within 3 months prior to the strain injury, smoking, type I or type II diabetes, and rheumatic diseases or organ dysfunctions. The considerable number of participants dropping out of this study (*n* = 15) was directly related to repeated COVID‐19 lockdowns imposed on Danish citizens. The study was conducted according to the Declaration of Helsinki, approved by The Regional Ethical Committee (the Capital Region of Denmark, ref. H‐19027293) and registered at clinicaltrials.gov (NCT04100161). All participants gave written informed consent to participate. Study data were collected and managed using REDCap electronic data capture tools (Harris et al., 2009; Harris et al., 2019). Participants were randomized to receive either a protein or placebo supplement; however, no difference was observed between the groups (see Mertz et al. (2025) for details) and therefore they were combined in the present study.

### 2.2 MR imaging and quantification

**FIGURE 1:**
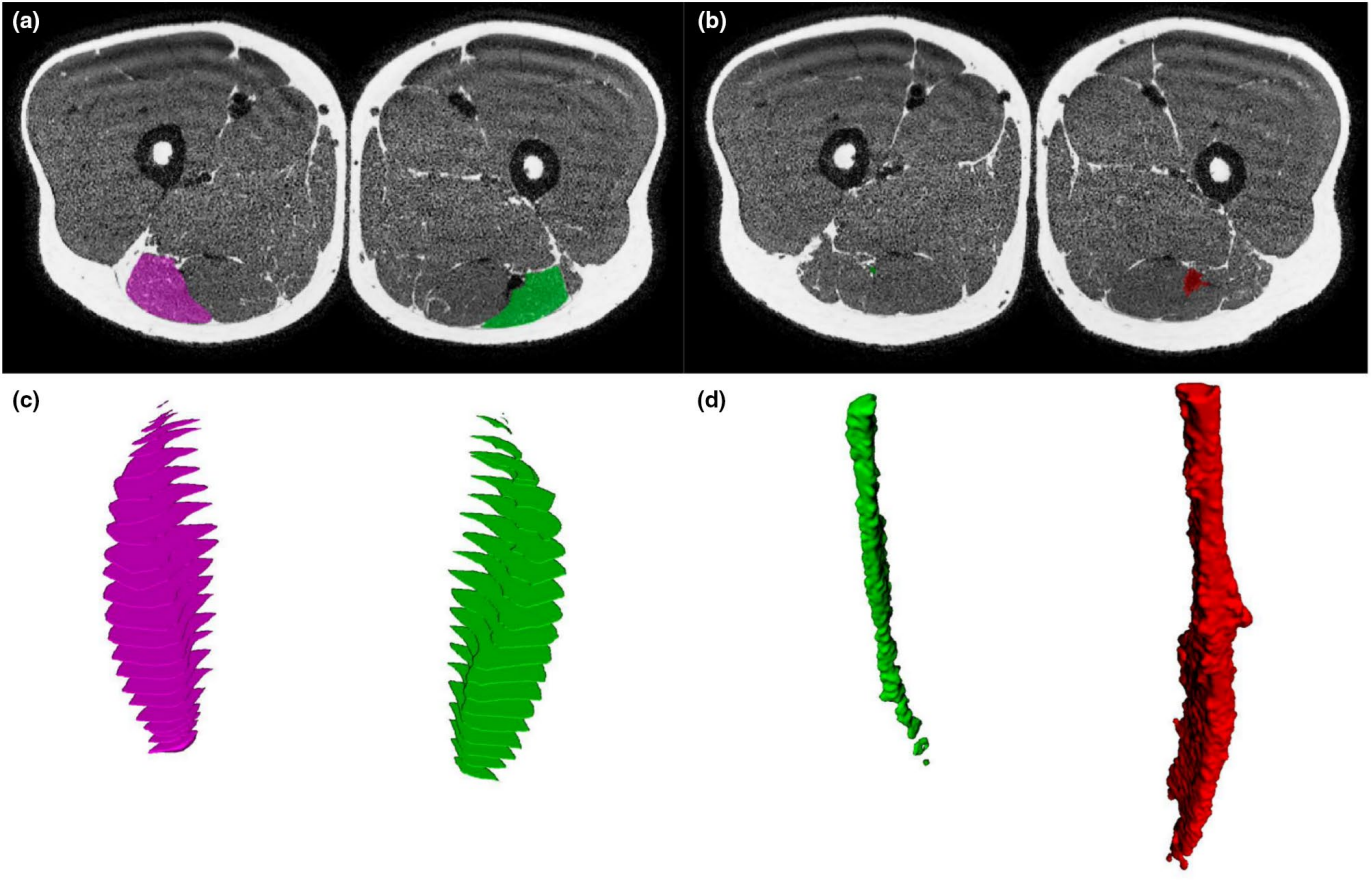
Representative image of a transverse isotropic 3D T1‐weighted sequence (a). Segmentation indicates ROIs from muscle volume measurements (Mertz et al., 2025) in the injured (green color ROI) and uninjured (purple color ROI) biceps femoris long head 3 months post injury (T2) (c). The same ROIs were transferred to DIXON images for muscle fat fraction quantification. Representative image of ROIs from aponeurosis volume measurements in the injured (red color ROI) and uninjured (green color ROI) (Mertz et al., 2025) (b). Three‐dimensional representation of the aponeurosis segmentations ROIs (d).

**FIGURE 2:**
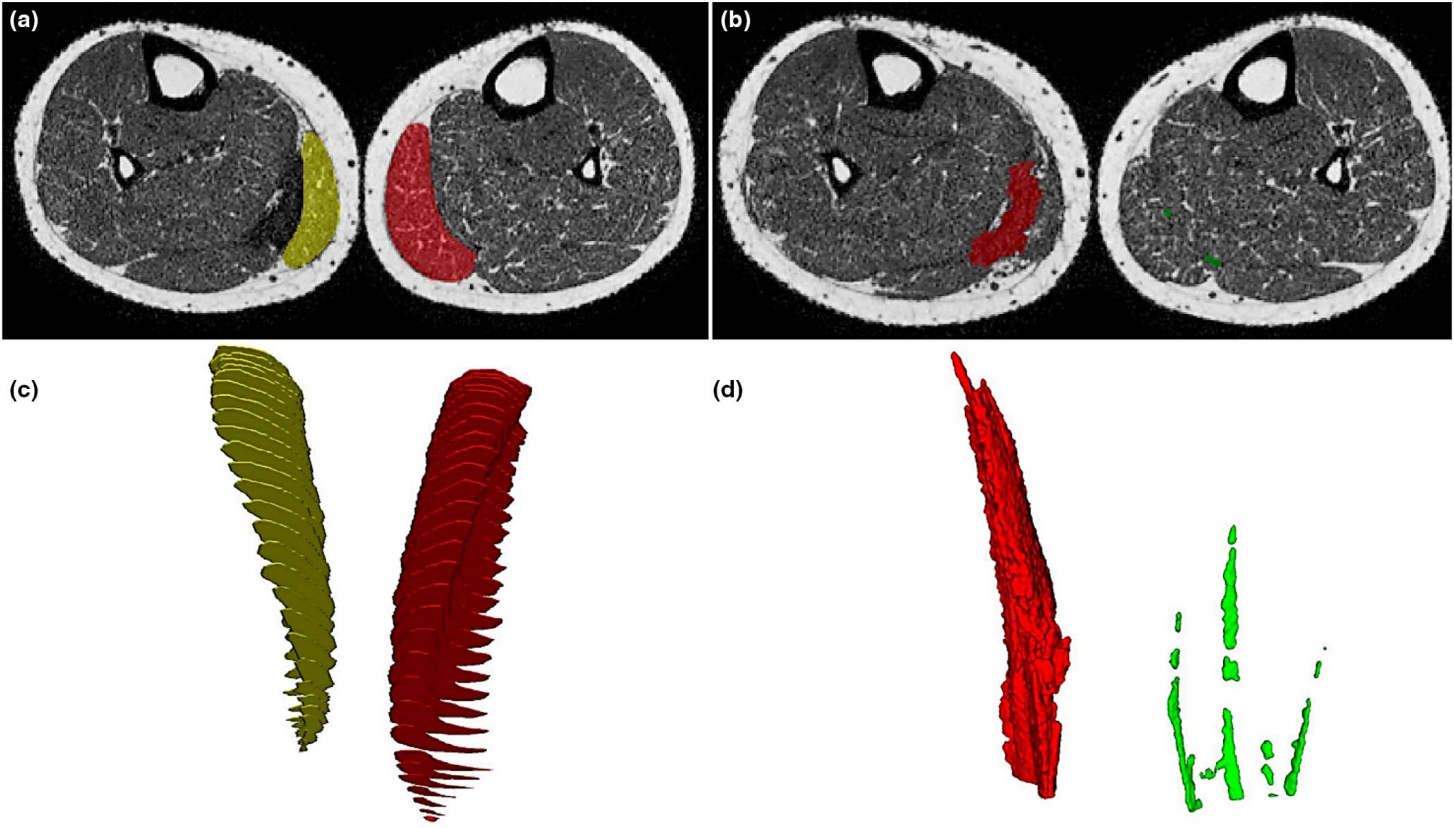
Representative image of a transverse isotropic 3D T1‐weighted sequence (a). Segmentation indicates ROIs from muscle volume measurements (Mertz et al., 2025) in the injured (yellow color ROI) and uninjured (red color ROI) gastrocnemius medialis 3 months post injury (T2) (c). The same ROIs were transferred to DIXON images for muscle fat fraction quantification. Representative image of ROIs from aponeurosis volume measurements in the injured (red color ROI) and uninjured (green color ROI) (Mertz et al., 2025) (b). Three‐dimensional representation of the aponeurosis segmentations ROIs (d).

All patients were MRI‐scanned (Philips Ingenia Ambition 1.5T scanner, software version 5.6.1.2, Netherlands) in the first week after the injury (mean days after injury ± SD; 7.3 ± 2.6 days), 3 months (after the rehabilitation period, at least 48 h following the last training session), and again 12 months post injury. The participants were scanned in a supine position feet first, with their feet placed against a plastic foot plate at approximately 30° plantarflexion. Axial 6‐point DIXON‐Quant sequences were acquired with the patient in a feet‐first supine position, using a spine coil posteriorly and a body coil anteriorly. Imaging parameters were as follows: repetition time (TR) 5.3 ms (shortest), echo time (TE) 0.92 ms (shortest), field of view (FOV) 400 × 350 mm (RL × AP), slice thickness 6 mm, 77 slices in 2 stacks, pixel size (2 mm/pixel), flip angle 5°, and echo train length 6. DIXON reconstructions included water, fat, fat fraction, and T2* images. Parallel imaging was applied using SENSE. A transverse isotropic 3D T1‐weighted sequence (TE = 32 ms, TR = 400 ms, voxel size 0.7 × 0.7 × 0.7 mm^3^) of the calf (calf strain) or hamstrings (hamstring strain) was recorded and used to measure muscle and aponeurosis volumes (Mertz et al., 2025). ITK‐SNAP open‐source (ITK snap version 3.8.0) was used for manual muscle segmentation and volume measurement (Yushkevich et al., 2006), Figures 1a,c and 2a,c, trained personnel performed the measurements. For manual muscle segmentation, as described in (Mertz et al., 2025), bony landmarks were used to ensure the exact same length at the acute scan (T1), the 3‐ (T2) and 12‐month (T3) post injury scans. For orientation on calf scans, the epiphyseal plate of the fibula head was taken; for hamstring scans, the epiphyseal plate of the femur head was taken. Further, for calf scans, segmentation started at the distal site on the first slice on which the medial gastrocnemius was visible and continued proximally to the last slice on which the medial gastrocnemius still was visible. For hamstring scans, segmentation started at the proximal site on the first slice on which the semitendinosus was visible and continued distally to the last slice on which the semitendinosus still was visible. For aponeurosis volume measurements (Figures 1b,d and 2b,d), the semiautomated active contour segmentation tool (ITK snap version 3.8.0.) was applied with a pre‐determined threshold for all subjects and time points. Both the injured and healthy legs were analyzed, Figures 1 and 2. For the hamstring strain injuries, the biceps femoris long head or the semitendinosus were analyzed depending on the location of the injury. The location of the injury was determined as previously described (Mertz et al., 2025). For the calf strain injuries, the injured muscle was the gastrocnemius medialis. Six participants were excluded from fat fraction analyses due to technical difficulties (artifacts in DIXON scans or extensive movement between DIXON and 3D scans).

### 2.3 Fat fraction analysis and data processing

To quantify the muscle fat content, DIXON data were upscaled and reoriented to match the 3D sequence using Fiji/ImageJ2 (Version 2.3.0) (Schindelin et al., 2012) with the Bio‐Formats (Linkert et al., 2010) and TransformJ (Meijering et al., 2001) plugins. The regions of interest (ROIs) from the muscle volume measurements, Figures 1 and 2, were used for extracting DIXON fat fraction in the injured and uninjured muscle. Due to a blooming effect of the high subcutaneous fat signal (Figure S4), the outer 7 voxels (5 mm) around the ROI were excluded to ensure that only intramuscular fat was evaluated. Fat fraction assessed from the DIXON data correlated well with fat fraction measured on T1‐weighted MRI scans (Akima et al., 2016; Ornowski et al., 2024), Figure S5. We extracted the mean fat fraction from each muscle volume instead of the median because the nature of fatty infiltration is typically focal with streaks of fat occurring inside the muscle. Since the majority of the volume is made up of non‐fat infiltrated muscle, the median values would not capture these infiltrations very well (Figure S6).

### 2.4 Statistics

Data are presented as median and 95% confidence intervals (C.I.), unless otherwise stated. Overall effects of injury (between‐leg differences) and time and their interactions were analyzed by repeated measures mixed‐effects analysis. In case of significant injury × time interactions, Dunnett's multiple comparison test was applied. Simple linear regressions were applied to explore associations between changes in fat fraction as a function of the change in muscle volume or as a function of the magnitude of the structural injury calculated as the difference in aponeurosis volume between uninjured and injured muscle. Changes were evaluated from the baseline (acute) scan to the 3 months follow up scan and from baseline to the 12 months follow up scan in the injured muscle. Aponeurosis volume was only evaluated at 3 and 12 months follow up, not at baseline, due to the nature of the injury with extended hematoma acutely which could falsely estimate the aponeurosis volume. Statistics were performed in GraphPad Prism version 10.2.3 for Windows (GraphPad Software, San Diego, CA, USA).

## 3 RESULTS

**FIGURE 3:**
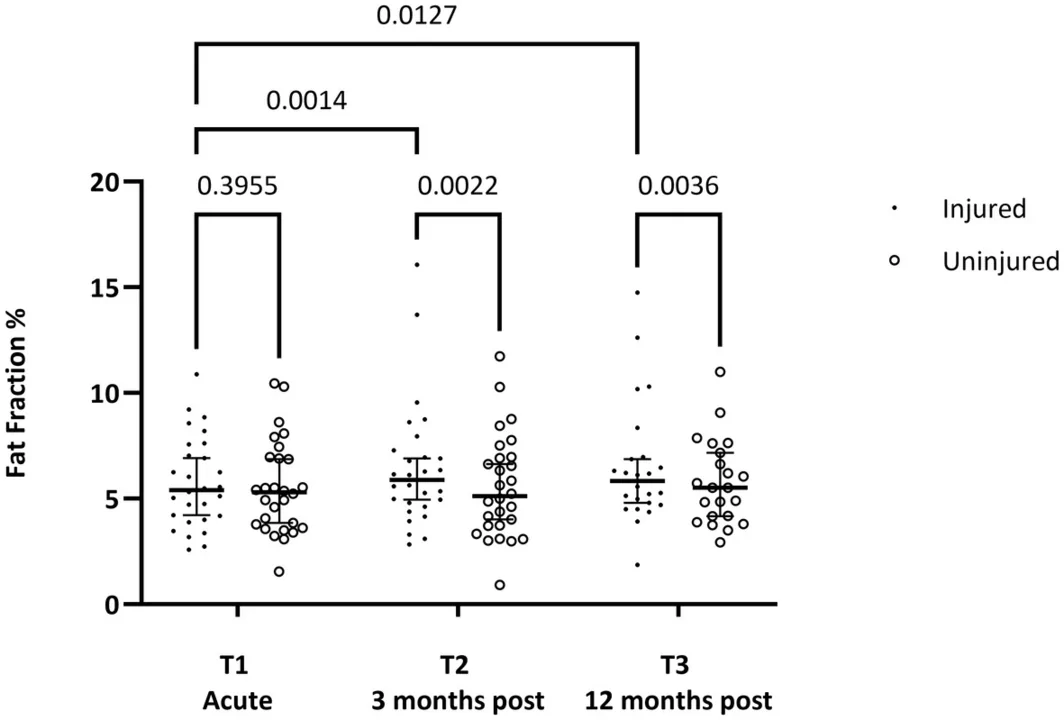
Fat fraction (%) in the injured and uninjured muscles determined acute (T1), 3 months post (T2) and 12 months (T3) post injury. Filled circles: Injured leg, open circles: Uninjured leg. Lines at median ± 95% C.I.

A time × injury interaction was observed for the fat fraction (*p* = 0.01). Post hoc analyses showed no difference between injured and uninjured acutely after the injury; however, the injured muscles displayed a significantly higher fat fraction compared to the uninjured 3 months post injury (*p* = 0.002) and 12 months post injury (*p* = 0.004). The fat fraction in the uninjured leg remained stable over the 1‐year period with no differences between the time points, while the injured leg displayed a significant increase from baseline to both 3 months (*p* = 0.001) and 12 months post injury (*p* = 0.01), Figure 3. Figure S1 provides visualization of paired data points (injured –uninjured) and Figure S2 injured data points over time.

**FIGURE 4:**
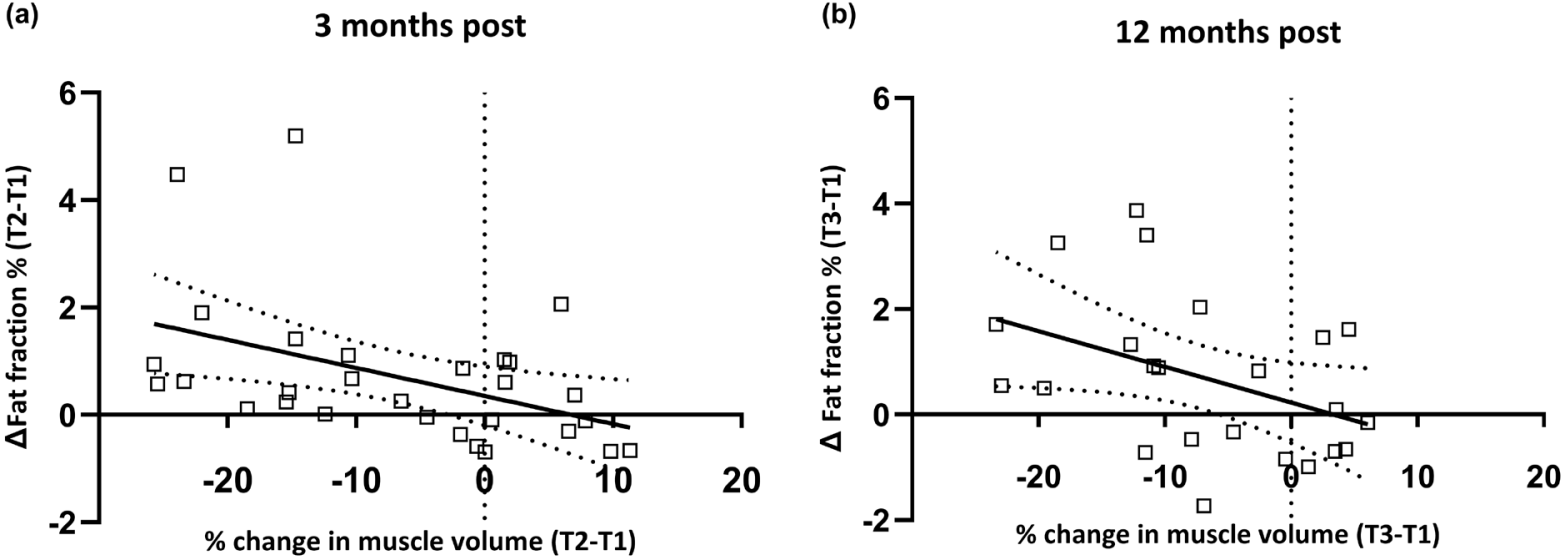
Relationship between the change in fat fraction and change in muscle volume from acute after the injury (T1) to 3 months post (T2) (a), and acute after the injury to 12 months post (T3) (b).

Simple linear regression showed a negative correlation between the percentage of change in muscle volume from acutely after the injury to the 3‐month follow up scan and the percentage of change in fat content from the acute to the 3‐month post scan (*r* ^2^ = 0.2, *p* = 0.02). This relationship indicates that the more muscle mass is lost, the higher the fat content in the muscle, Figure 4a. The association between the change in muscle volume from acute to 12 months and the change in fat content from acute to 12 months was not significant (*r* ^2^ = 0.17, *p* = 0.05), Figure 4b.

Next, we asked whether the observed aponeurosis enlargement, as a measure of the magnitude of structural injury on the connective tissue, would be associated with the increase in fat fraction from the acute time point to the 3 months follow up scan and to the 12 months follow up scan. The underestimate of the uninjured aponeurosis volumes due to insufficient imaging resolution to capture the thinnest regions (Figure 2d) adds uncertainty to this comparison, emphasizing the need for further validation of this exploratory analysis. Simple linear regression of the relative difference in aponeurosis volume in the injured compared to the uninjured aponeurosis 3 months post and the change in fat content from the acute to the 3 months post scan indicated a positive correlation (*r* ^2^ = 0.5, *p* < 0.01), Figure S3A. The output of the linear regression analysis of the aponeurosis enlargement at the 12 months post scan and the change in fat fraction from acute to 12 months post injury revealed a *p* value of 0.2, *r* ^2^ = 0.1, Figure S3B.

## 4 DISCUSSION

This observational study is the first to quantify fat content in the whole muscle volume after a strain injury and reveals two key findings: (1) There was a significant overall increase in fat fraction in the injured muscle 3 months after the injury. This rise in fat content appears stable as fat fraction remained elevated 12 months after the injury in the injured muscle compared to the uninjured muscle and compared to the fat content in the injured muscle acutely after the injury. Fat fraction of the uninjured muscle remained stable over the 1‐year period. (2) Greater loss of muscle volume was correlated to increased fat content at 3 months and also the extent of the aponeurosis volume enlargement potentially correlated with increased fat content at 3 months.

To our knowledge, this is one of few data sets showing fatty infiltration in human skeletal muscle relatively early after a trauma. Gorgey and Dudley (2007) compared a group of patients with an incomplete spinal cord injury to an age‐matched control group and reported increased intramuscular fat in the thigh approximately 6 weeks and 4–5 months post injury. Fatty infiltration in the muscle has been detected in individuals 1 year (Hoeffner et al., 2024) or longer (Eken et al., 2021) after an Achilles tendon rupture. In degenerative conditions such as end‐grade hip osteoarthritis (Fitzgerald et al., 2023), or rotator cuff tears (Kriechling et al., 2025; Lacheta et al., 2023; Wieser et al., 2019), fat infiltration probably develops over a prolonged time. In addition, we observe a stable increase in fat as there was no improvement or further increase at the 1 year follow up MRI scan. This should be seen in the light of all involved individuals having returned to full participation in sports (median days from injury to return to sport 48 ± 18) (Mertz et al., 2025). Despite active sports participation and loading of the musculoskeletal system, fat content following muscle strain injury does not normalize to uninjured levels. Similarly, the muscle volume lost after injury is not regained either.

A great number of scientific studies investigating fatty infiltration in skeletal muscle focus on muscular changes following rotator cuff tears, which often occur in individuals around the age of 60 (Hamano et al., 2017; Kriechling et al., 2025; Lacheta et al., 2023). Our cohort is a group of relatively young, sports‐active individuals with no other known conditions than their muscle strain injury (Mertz et al., 2025). Noteworthy, rotator cuff muscles are located in the upper extremities where the extent of immobilization following injury might be even greater than in leg muscles. Muscles in the thigh and lower leg are to a certain degree activated even in the early phase after injury as a part of locomotion. In addition, all subjects in the present cohort began rehabilitation 48 h post injury (Mertz et al., 2025), so the unloading phase of the injured tissues was limited. Nevertheless, we report a similar negative association between the loss of muscle volume and the fat fraction as in shoulder pathologies (Kriechling et al., 2025).

One explanation for the similar pattern in upper and lower extremities might be the defect in the interface between the force‐generating muscle and the aponeurosis. Our results indicate an association between the extent to which the aponeurosis increases in volume and the rise in fat fraction from the injury to 3 months post injury; however, confirmation of these findings is required in future studies. This finding suggests a coupling between the changes in the aponeurosis where muscle fascicles insert and the pathologic changes within the muscle per se. We have previously shown that the site at which the muscle fascicles insert into the aponeurosis is severely disturbed after a strain injury: Ultrasound imaging suggests that fascicles at site of the injury are not contracting, but rather passively pulled along by the aponeurosis (Nielsen et al., 2023). Recent observations by Khair et al. support the note that muscular adaptations following tendon injury are at least partly driven by alterations of the aponeurosis properties, or the other way around (Khair et al., 2025). It means that a change in forces exerted by muscle fascicles at the aponeurosis insertion impacts the aponeurosis structure and function, or that an aponeurosis with altered mechanical properties can lead to pathologic changes within the muscle. This link and its direction are speculative, but the data presented here in combination with previous findings on the aponeurosis after strain injuries supports the hypothesis that the muscle‐aponeurosis/tendon unit after a strain injury is not sufficiently repaired and that this persisting defect might have a direct impact on muscle quality.

It is currently unknown whether affected muscle fascicles at the site of a strain injury remain innervated or are unable to generate contractile force or a combination thereof. Preliminary data based on electron microscopy of biopsies from chronic strain injured muscles demonstrate a loss of contractile elements (Bayer et al., 2021) and the current data support the notion that muscle quality has become poorer observable as fat accumulation in the muscle. A previous study applying conventional surface EMG reported lower activation of hamstring muscles in persons with a history of hamstring strains, especially in the eccentric phase, suggesting persistently altered muscle activation following a strain injury (Presland et al., 2021). A significant reduction in muscle contractility might explain the irreversible nature of fatty infiltration following injuries, trauma, and surgery as the muscle itself cannot generate tension at the same level as pre‐trauma. Consequently, changes in the biomechanical cues within the muscle tissue and the associated aponeurosis might have an impact on the cells residing in muscle tissue. Fibro/adipogenic progenitors (FAPs) are a heterogeneous group of cells which can undergo adipogenic or fibrogenic differentiation and have been associated with fatty infiltration in experimental animal models (Joe et al., 2010; Uezumi et al., 2010). The cellular source and processes involved in fatty infiltration of human skeletal muscle following trauma are poorly understood, although some data from human FAPs in pathologic muscles are emerging (Fitzgerald et al., 2023). The dynamics, the heterogenicity of FAPs and interplay with other cell types following muscle strain injuries remain, however, to be explored.

The present study quantifies fat fraction of the entire injured muscle volume. This approach contrasts with most other studies, measuring fat content in just a single or few axial slices, which has been shown not to be predictive of fat fraction in the entire muscle volume (Vidt et al., 2016). While it is a strength to study the entire 3D muscle volume, fat fraction analysis at different regions of the muscle would have been interesting since, hypothetically, the increase in fat content after a strain injury could be markedly higher at the site of the muscle where the injury occurred. However, analyzing fat content in a small region like that is technically challenging and was not possible with the present MRI scans, in part due to the lower resolution of the Dixon scans (Figure S4). Further, in lieu of biopsies or more advanced MRI spectroscopic methods, the present measures of fat fraction by Dixon MRI represent our best estimates, but it should be noted that assumptions underlying these measurements may be challenged at the low levels of fat content observed in the present study (Roberts et al., 2018). Finally, the aponeurosis forms a continuous sheet; however, in the uninjured limbs, the imaging resolution was insufficient to capture the thinnest regions as is evident from Figure 2d. As a result, our aponeurosis volumes on the uninjured side are slightly underestimated; however, we chose to include these results to provide some numerical comparators.

## 5 CONCLUSION

We document a significantly higher fat fraction following a strain injury in otherwise healthy, sports‐active individuals, occurring within the first 3 months post injury. The elevated fat content does not appear to normalize over time as fat content in the injured muscle is increased 1 year after the injury. At 3 months the increase in fat content was associated with the loss of muscle volume and enlargement of the aponeurosis. The latter observation emphasizes the importance of a functional coupling between the muscle tissue and the associated connective tissue, which does not seem to be re‐established following a strain injury. The underlying cellular processes which could explain the increase in fat following this type of injury are not yet understood.

## AUTHOR CONTRIBUTIONS

**Monika Lucia Bayer:** Conceptualization; data curation; formal analysis; funding acquisition; investigation; methodology; project administration; resources; supervision; validation; visualization. **Kenneth Hudlebusch Mertz:** Conceptualization; data curation; formal analysis; investigation; methodology; project administration. **Asta Skovgaard Eriksen:** Formal analysis; methodology; software. **Michael Kjaer:** Conceptualization; data curation; funding acquisition; investigation; supervision. **Stig Peter Magnusson:** Conceptualization; funding acquisition; investigation; supervision. **Frederik Hvid Linden:** Data curation; formal analysis. **Rene Brüggebusch Svensson:** Conceptualization; data curation; formal analysis; investigation; methodology; project administration.

## FUNDING INFORMATION

The authors disclosed receipt of the following financial support for the research of this article: Team Denmark (SPM), Novo Nordisk Fonden (Novo Nordisk Foundation), NNF (MK) (NNF22OC0079373), and Kulturministeriet (The Danish Ministry of Culture) (MLB) (FPK.2019‐0049).

## CONFLICT OF INTEREST STATEMENT

The authors have declared that there are no conflicts of interest in the authorship and publication of this contribution.

## ETHICS STATEMENT

Ethical approval for this study was obtained from The Regional Ethical Committee, ref. H‐19027293 and registered at clinicaltrials.gov (NCT04100161).

## Supporting information


Appendix S1.

## Data Availability

The data that support the findings of this study are available upon request from the corresponding author. The data are not publicly available due to privacy or ethical restrictions.
