# Supplementary material for: Early and persisting increase in fat content after human muscle strain injuries in adults
Source: Physiol Rep. 2026 Sep 1;14(17):e71086. doi: 10.14814/phy2.71086 (PMC13535331; doi:10.14814/phy2.71086)
Supplement: Supplementary file 1 [file PHY2-14-e71086-s001.docx]

Early and persisting increase in fat content after human muscle strain injuries in adults

|  | Protein  N = 13 | Placebo  N = 16 |
| --- | --- | --- |
| **Demographics** |  |  |
| *Age (years)* | 39.8 ± 10.45 | 41.0 ± 11.6 |
| *Males/females* | 12/1 | 13/3 |
| *BMI (kg/m^2^)* | 25.7 ± 2.9 | 25.4 ± 2.0 |
| **Injury site** |  |  |
| Hamstring (N) | 3 | 4 |
| Calf (N) | 10 | 12 |

**Supplementary files**

**Table S1.** Baseline characteristics of the included participants. Data are presented as means and standard deviations. Adapted from Mertz et al ^2^**.**

**Supplementary figure S1.** Fat fraction (%) in the injured and uninjured muscles determined acute (T1), 3 months post (T2) and 12 months (T3) post injury. Filled circles: injured leg, open circles: uninjured leg, ns = non-significant, * p< 0.05, ** p< 0.01. Lines connect paired data points (injured – uninjured muscle).

**Supplementary figure S2.** Fat fraction (%) in the injured muscles over the three time points: Acute (T1), 3 months post (T2) and 12 months (T3) post injury.

**Supplementary figure S3.** Region of interest before removal of the outer 7 voxels (5 mm) on axial T1w-3D image (A), DIXON fat image (B), DIXON fat fraction (C), and DIXON water (D). The DIXON images (B, C, D) are upscaled to match the T1w-3D image and ROI. It is clear that some signal from the surrounding subcutaneous fat overlaps with the muscle ROI (B, C). ROI shows correct placement on medial gastrocnemius muscle (A). Bottom row shows original (not upscaled) DIXON fat (E), water (F) and fat fraction (G) from approximately the same axial section as A-D.

**Supplementary figure S4**. Relationship between fat fraction measured from Dixon versus the intensity of T1 weighted images (“Otsu2”) using a similar approach as previously reported ^1,3^. The original Otsu method is described here: (<https://ieeexplore.ieee.org/stamp/stamp.jsp?tp=&arnumber=4310076>). Note that this plot only includes fat fraction analysis of the injured and uninjured gastrocnemius medialis muscles and not hamstrings.

**Supplementary figure S5**. Example histograms of fat fraction in the injured and uninjured gastrocnemius muscle of one participant. A) Histogram showing % of voxels with a given fat fraction. B) Same data on a logarithmic scale for illustration of the tail. Bin size = 1% fat fraction.

**References**

1.	Akima H, Hioki M, Yoshiko A, et al. Intramuscular adipose tissue determined by T1-weighted MRI at 3 T primarily reflects extramyocellular lipids. *Magn Reson Imaging*. 2016;34(4):397-403.

2.	Mertz KH, Sorensen HH, Lorentz C, et al. Effects of Protein Supplementation During Early Rehabilitation on Muscle Volume and Function After Acute Muscle Strain Injuries: A Randomized Controlled Trial. *Scand J Med Sci Sports*. 2025;35(4):e70043.

3.	Ornowski J, Dziesinski L, Hess M, et al. Thresholding approaches for estimating paraspinal muscle fat infiltration using T1- and T2-weighted MRI: Comparative analysis using water-fat MRI. *JOR Spine*. 2024;7(1):e1301.
